# Growth Efficiency of *Chlorella sorokiniana* in Synthetic Media and Unsterilized Domestic Wastewater

**DOI:** 10.3390/biotech12030053

**Published:** 2023-08-03

**Authors:** Svetlana S. Bulynina, Elvira E. Ziganshina, Ayrat M. Ziganshin

**Affiliations:** Department of Microbiology, Institute of Fundamental Medicine and Biology, Kazan (Volga Region) Federal University, 420008 Kazan, Republic of Tatarstan, Russia; svesbulynina@kpfu.ru (S.S.B.); elvira.ziganshina@kpfu.ru (E.E.Z.)

**Keywords:** *Chlorella sorokiniana*, photobioreactor, synthetic media, domestic wastewater, nutrient recycling, proteins, bacterial community structure

## Abstract

Incorporating a variety of microalgae into wastewater treatment is considered an economically viable and environmentally sound strategy. The present work assessed the growth characteristics of *Chlorella sorokiniana* during cultivation in balanced synthetic media and domestic wastewater. Increasing the NH_4_^+^–N concentration to 360 mg L^−1^ and adding extra PO_4_^3−^–P and SO_4_^2−^–S (up to 80 and 36 mg L^−1^, respectively) contributed to an increase in the total biomass levels (5.7–5.9 g L^−1^) during the cultivation of *C. sorokiniana* in synthetic media. Under these conditions, the maximum concentrations of chlorophylls and carotenoids were 180 ± 7.5 and 26 ± 1.4 mg L^−1^, respectively. Furthermore, when studying three types of domestic wastewaters, it was noted that only one wastewater contributed to the productive growth of *C. sorokiniana*, but all wastewaters stimulated an increased accumulation of protein. Finally, the alga, when growing in optimal unsterilized wastewater, showed a maximum specific growth rate of 0.73 day^−1^, a biomass productivity of 0.21 g L^−1^ day^−1^, and 100% NH_4_^+^–N removal. These results demonstrate that the tested alga actively adapts to changes in the composition of the growth medium and accumulates high levels of protein in systems with poor-quality water.

## 1. Introduction

Microalgal biomass is a multifunctional product that plays an important role in the development of the modern bioeconomy. Existing microalgae production technologies focus on waste recycling, reducing water and energy consumption, and securing a competitive advantage for algal biomass among different feedstocks. For the successful introduction of algal metabolites into large-scale processes, it is necessary to develop appropriate biotechnologies that provide a large amount of biomass [1].

One of the options in this case is the use of photobioreactors that compensate for the shortcomings of open cultivation systems [2]. The efficiency of cultivation in closed systems depends on the experimentally established optimal values of the main parameters that affect the biomass yield and biochemical composition of cells [3,4]. At present, various types of photobioreactors have been developed that provide uniform distribution of light and regulation of its intensity, dosing of CO_2_ and O_2_, maintaining the required pH value of the medium, mixing, and aseptic conditions [5]. Cultures grown under mixotrophic and heterotrophic conditions are more sensitive to bacterial contamination (compared to photoautotrophic cultures) since they grow in the presence of various sources of organic carbon [6]. While a photobioreactor can provide complete sterility, in large-scale processes, maintaining strict aseptic conditions and sterilizing culture media increase culture costs. Therefore, it is also important to consider the use of non-sterile culture media.

To increase the profitability of cultivation systems, various waste streams can be used as alternative sources for the macro- and microelements necessary for the growth of algal cultures [7]. In recent years, both domestic and agricultural wastewaters have been considered accessible sources of water and nutrients. The main problems associated with the implementation of this approach are the wide variety of chemical and physical properties of wastes and the need for their pretreatment [8,9]. The composition of nutrient media for microalgal growth is determined by the physiological characteristics of strains and must be adapted to a specific cultivation regimen [10]. The key points in optimizing cultivation regimens are carbon exchange, nitrogen, phosphorus, and sulfur uptake [11,12].

Photosynthetic microorganisms are very efficient at capturing CO_2_ and therefore make a significant contribution to the development of carbon-neutral technologies. By fixing CO_2_, algae synthesize valuable biomolecules [13], but the cultivation efficiency may decrease if the CO_2_ concentration deviates significantly from the optimal one. It has been established that the level of CO_2_ affects the specific growth rate, biomass yield, and efficiency of nutrient removal by algal cells [14]. Mixotrophic cultivation makes it possible to use different carbon sources and reduce the influence of limiting factors that accompany autotrophy or heterotrophy [15,16]. The efficiency of organic carbon assimilation largely depends on the composition of the media as well as on the cultivation regimen [11].

The cultivation conditions in a photobioreactor also require the use of artificial light sources, such as fluorescent lamps or light-emitting diodes (LED). In experiments on the effect of light exposure on the productivity of microalgae, various photoperiods (e.g., 12:12, 14:10, 16:8, 24:0-h light/dark cycles) were tested [17,18,19]. It has been shown that a longer period of darkness can reduce the productivity of algae as it inhibits the functioning of the cell photosystems [20]. The optimal light/dark cycle determines the algal culture growth rate, total biomass yield, accumulation of proteins, lipids, and carbohydrates, as well as the uptake of macro- and microelements [21,22].

The productivity of microalgae increases with a sufficient content of nitrogen and phosphorus in the nutrient medium. In addition, the concentration of these macronutrients affects the biochemical composition of cells, which is of great importance in the production of some value-added products [23]. For example, a decrease in nitrogen levels leads to a decrease in protein yield [4] but stimulates the synthesis of other cellular components [24,25]. Data indicate that nitrogen deficiency is one of the main limiting factors in biomass accumulation [17,26]. In the process of assimilation of NH_4_^+^, microalgae save energy since this form of nitrogen is used directly by cells in contrast to NO_3_^−^ [27,28]. Under certain conditions, such as elevated temperature and pH values, a fraction of NH_3_ increases, which is toxic to many organisms. For this reason, when cultivating algae in wastewater with a high content of ammonium nitrogen, only strains with a high tolerance for this factor are used [11,28]. Among organic forms of N, urea is an available and cheap nutrient [29], constituting a significant proportion of nitrogen compounds in domestic wastewater [30]. The need for algae in P can be determined by the physiological characteristics of the strain, the method of cultivation, and the presence of other nutrients [31]. Successful use of green algae (including *Chlorella* species) in various biotechnologies is possible due to their cultural features. The competitive advantage lies in the fact that *Chlorella* species are characterized by relatively high growth rates and the accumulation of valuable metabolites [4].

The first aim of the work was to evaluate the growth characteristics of *Chlorella sorokiniana* in synthetic media at different photoperiods and macronutrient supplies. The complex analysis of the treatment of various domestic wastewaters in original and diluted forms by microalga, with a simultaneous assessment of the bacterial load in cultivation systems, is the second goal of the presented work. Finally, the current work considers efficient strategies for cultivation of green algae, which reflect the possibilities and limitations of the bioremediation of wastewater by potential microalgal strains.

## 2. Results and Discussion

### 2.1. Growth Characteristics of Chlorella sorokiniana in the Synthetic Media

In the present study, microalga was cultivated under photoautotrophic growth regimens in a modified Bold’s Basal Medium (BBM) in which the only source of nitrogen was ammonium chloride. Cultivation was carried out in a 6.8 L glass culture vessel with a working volume of 5.0 L using LED full spectrum lamps as a lighting source. In the first case, the nitrogen concentration in the nutrient medium corresponded to a concentration of 180 mg L^−1^, while in the other series of experiments, the nitrogen level was increased by 2 times to reach 360 mg L^−1^. To assess the role of the N/P/S ratio, microalgae were additionally supplied with different concentrations of phosphorus and sulfur. Another factor assessed in this study was the photoperiod, the effect of which was determined at 16:8 and 24:0-h light/dark cycles (Table 1).

The maximum level of biomass at a photoperiod of 16:8 (16 h of light, 8 h of darkness) and a nitrogen supply of 180 mg L^−1^ was 3.17 ± 0.13 g L^−1^ (SM1), while under continuous lighting it was higher and reached 4.17 ± 0.14 g L^−1^ (SM2) (Table 1). The increased content of P and S in the medium made it possible to achieve a slightly higher level of biomass, which amounted to 4.40 ± 0.17 g L^−1^ (SM3), but these data were statistically insignificant (when SM2 and SM3 treatments were compared). The increase in NH_4_^+^–N concentration to 360 mg L^−1^ (SM4) at standard levels of P and S led to higher biomass yield compared with SM2 and SM3 treatments, but these data were also statistically insignificant. This was probably associated with an imbalance in the main nutritional components. The strategy of adding extra P and S was a solution to this problem (SM6 and SM5), ultimately increasing the total biomass values (5.68–5.93 g L^−1^). Additionally, it was found that fractional nitrogen nutrition (SM6) led to less foaming in the reactor, but this regimen turned out to be slightly less effective compared to the initially increased concentration of NH_4_^+^–N (SM5).

**Figure 1:**
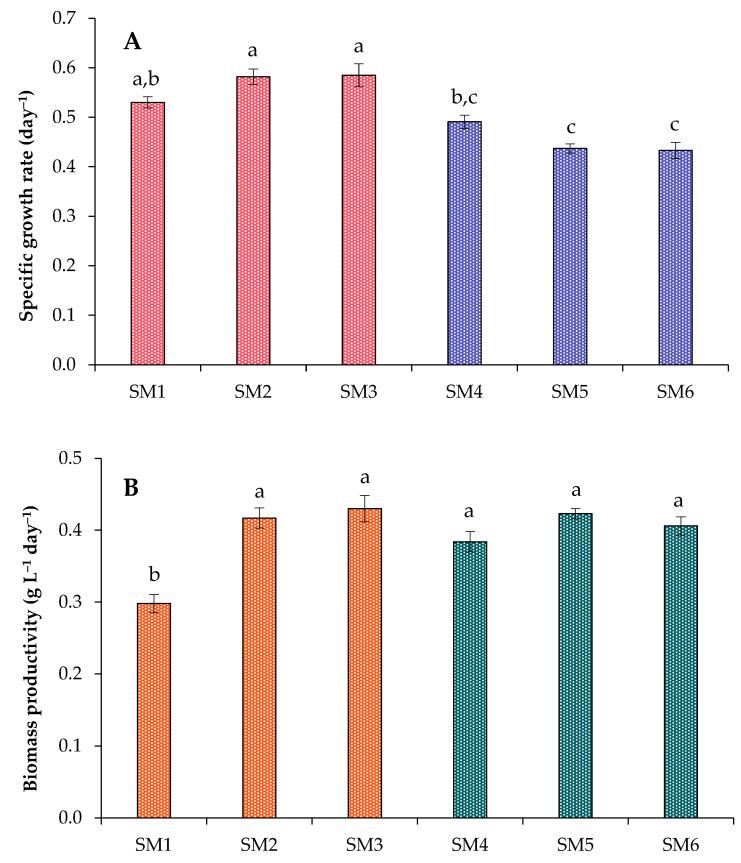
Specific growth rate (**A**) and biomass productivity (**B**) of *C. sorokiniana* grown in synthetic media under different strategies (SM1–6). Means that do not share a letter are significantly different.

Optical density values at 750 nm (OD_750nm_) and cell concentrations were additionally measured (data not shown). Key characteristics, such as specific growth rate and biomass productivity, were further defined to describe the culture’s growth kinetics (Figure 1A,B). As a result, all the studied factors had impacts on growth parameters. Although the specific growth rate was lower in SM5 and SM6 experiments compared with SM2 and SM3 experiments (probably due to high NH_4_Cl concentrations), the biomass productivity was the same in these experiments. Therefore, the combination of continuous lighting and balanced nutrition can lead to the highest final biomass yield, highlighting the possibility of obtaining more biomass without replacing large volumes of medium. This algal strain can also be applied to treat wastewater with high nutrient levels. When comparing the results obtained in SM1 treatments with the data obtained by us earlier using another type of photobioreactor (3.6 L Labfors 4 Lux (Infors HT, Bottmingen, Switzerland)), we noted similar data on total biomass yield for the same algal strain in the same growth medium, but specific growth rate and biomass productivity were lower [27]. This means that different designs of photobioreactors and light sources/intensities can have different effects on algal growth kinetics.

**Figure 2:**
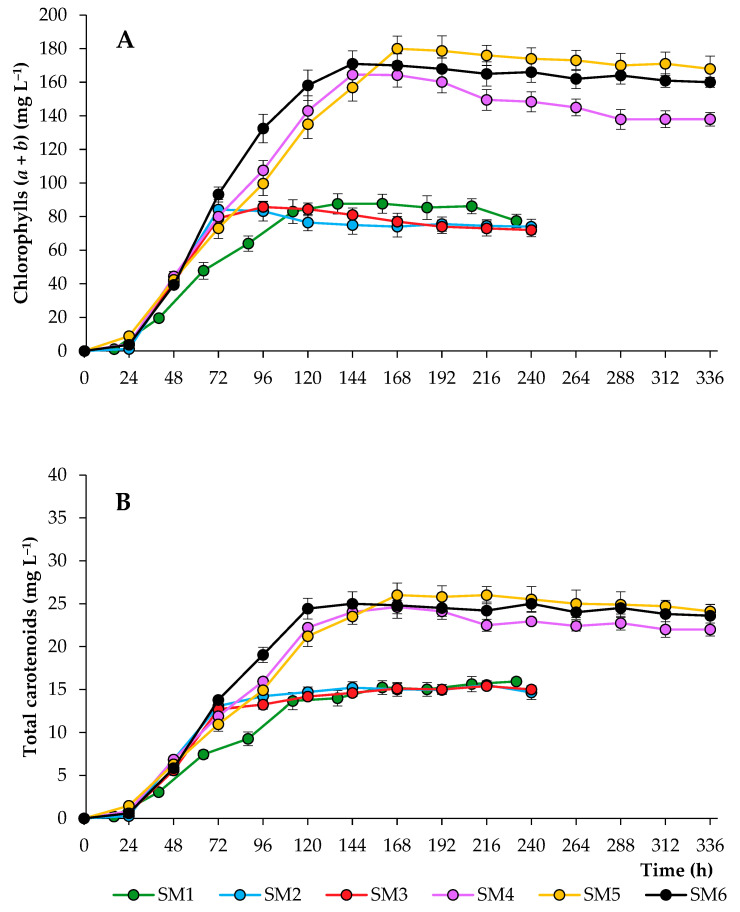
Chlorophyll (**A**) and carotenoid (**B**) concentrations of *C. sorokiniana* grown in synthetic media under different strategies.

The main pigments of algae with high commercial value are chlorophylls (*a*, *b*) and carotenoids (xanthophylls and carotenes). Due to their bioactive properties, pigments are widely used in the pharmaceutical and food industries [32]. The main factor influencing the accumulation of cellular pigments was the level of N in the medium (Figure 2). While the maximum concentration of total chlorophylls and total carotenoids was achieved in SM5 treatments (180 ± 7.5 and 26 ± 1.4 mg L^−1^ at 168 h, respectively), the highest rate of pigment synthesis was characteristic of fractional N nutrition (SM6), with a maximum level detected at 144 h (171 ± 7.7 and 25 ± 1.3 mg L^−1^, respectively). The growth of alga at a N supply of 180 mg L^−1^ led to a halving of the pigment content, regardless of the availability of P and S. It is important to note that a more intense synthesis of carotenoids was observed under continuous lighting. This can be explained by the fact that an important function of carotenoids is to ensure cell adaptation to light conditions, especially light of high intensity [33].

**Figure 3:**
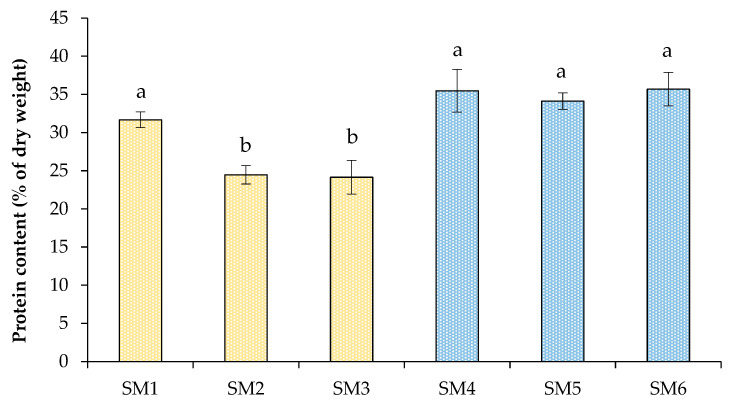
Protein content in *C. sorokiniana* cells grown in synthetic media under different strategies. Means that do not share a letter are significantly different.

Microalgal biomass is high in protein compared to several other plant and animal sources and has a rich amino acid composition. The accumulation of protein in cells depends both on the species of microalgae and on cultivation conditions, the change of which can achieve a more efficient production of proteins [4,34]. The protein content in the dry biomass of algal cells was determined at the end of cultivation (Figure 3); therefore, the differences can not only be associated with the growth conditions, but also with the cultivation time since an increase in the cultivation period can cause a change in the ratio of cellular metabolites. The protein content in algal cells in different treatments varied from 24% to 36%, with the highest values observed at elevated nitrogen levels (SM4–SM6). The accumulation of protein in cells was also affected by the duration of lighting (when comparing SM1 and SM2 treatments). In a study performed by Madhubalaji et al. [21], it was found that a 16:8-h light/dark cycle was useful in obtaining high concentrations of proteins and lipids, but a continuous lighting was suitable for increasing biomass productivity. In another study [22], both biomass productivity and protein/lipid content were higher at a photoperiod of 16:8-h. Nordin et al. [35] showed that nitrogen deficiency completely changed the ratio of the main valuable metabolites towards the accumulation of lipids and carbohydrates.

**Figure 4:**
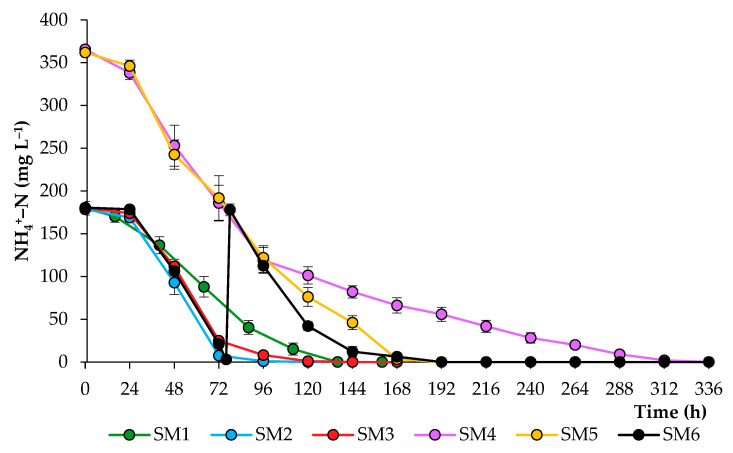
Ammonium nitrogen concentrations in synthetic media during the growth of *C. sorokiniana*.

An important indicator of a balanced nutrient medium and optimal growth conditions is the rate and degree of the consumption of nutrients. In this work, the consumption of NH_4_^+^–N by algal cells from the culture medium was monitored daily (Figure 4). Under tested cultivation conditions, 99% of the total ammonia was represented by ammonium ions. Continuous lighting contributed to faster N assimilation by algal cells, with complete removal at 96 h of cultivation in the SM2 treatments and at 136 h of cultivation in the SM1 treatments. In the case of a high N concentration in the nutrient medium (SM4–SM6), the presence of excess P and S played the greatest role in N assimilation by algal cells. This led to the complete removal of NH_4_^+^–N by cells within 168 h in SM5 and SM6 treatments and the prolongation of NH_4_^+^–N assimilation in SM4 treatments.

Species of the family *Chlorellaceae* are among the few microalgae recognized as suitable for large-scale use [36]. Within *Chlorellaceae*, *C. vulgaris*, *C. sorokiniana*, *Auxenochlorella pyrenoidosa*, *Auxenochlorella protothecoides*, and many others are deeply studied species providing high biomass yield and several metabolites [37]. Regardless of the species of microalgae and type of metabolism, optimal growth depends on the availability of nutrient sources, among which the main ones are N, P, and S. Recently, Zurano et al. [38] investigated the role of various nutrients and their concentrations on the growth of microalgae under photoautotrophic conditions. The authors found that both a deficiency and an excess of nutrients reduced the efficiency of metabolic processes. In addition, during photoautotrophic growth, important factors influencing the efficiency of the absorption of macro- and microelements are the C/N/P ratio and the light regimen, as shown by Arcila et al. [39]. In the process of assimilation of NH_4_^+^, microalgae use it directly, in contrast to NO_3_^−^, whose consumption depends on the activity of nitrate reductase [28]. Microalgae have different phosphorus uptake strategies. Under conditions of excess, phosphorus accumulates in cells in the form of polyphosphates, which provide a pool of available phosphorus in the absence of it in the external environment [11].

### 2.2. Growth Characteristics of Chlorella sorokiniana in Domestic Wastewaters

Wastewaters from three septic tanks, in their original and diluted forms, were used as growth media for alga. Cultivation was carried out under continuous lighting and CO_2_ supply. The growth of *C. sorokiniana* in wastewater was assessed by several parameters, including growth kinetics, final biomass yield, organic matter content in biomass, and the synthesis of pigments. For each type of wastewater tested, the initial content of the main components was determined.

Despite the different sources of domestic waters, the characteristics of all samples were within the following values: pH (7.6–7.8), total solids (0.19–0.32%), NH_4_^+^–N (90–130 mg L^−1^), NO_3_^−^–N (≤1 mg L^−1^), PO_4_^3−^–P (2–5 mg L^−1^), and SO_4_^2−^–S (≤1 mg L^−1^). Since wastewaters contained low concentrations of P and S, these elements, in the forms of H_3_PO_4_ and H_2_SO_4_, were additionally added to the original wastewaters to achieve the same P and S concentrations as in standard Bold’s Basal Medium. NH_4_^+^–N levels were also adjusted to achieve similar concentrations (up to 130 mg L^−1^). In diluted wastewater samples, N, P, and S levels were two times lower.

The biomass yield was consistently higher in all tests using undiluted wastewater. The maximum biomass value (1.26 ± 0.07 g L^−1^) was obtained when cultivating *C. sorokiniana* in wastewater from the third septic tank (WM3_i), and this value was 3–4 times higher compared to other treatments with undiluted wastewater (Table 2). Dilution of wastewaters led to a decrease in the amount of biomass, which was probably due to a lack of micronutrients for cell growth. 

**Figure 5:**
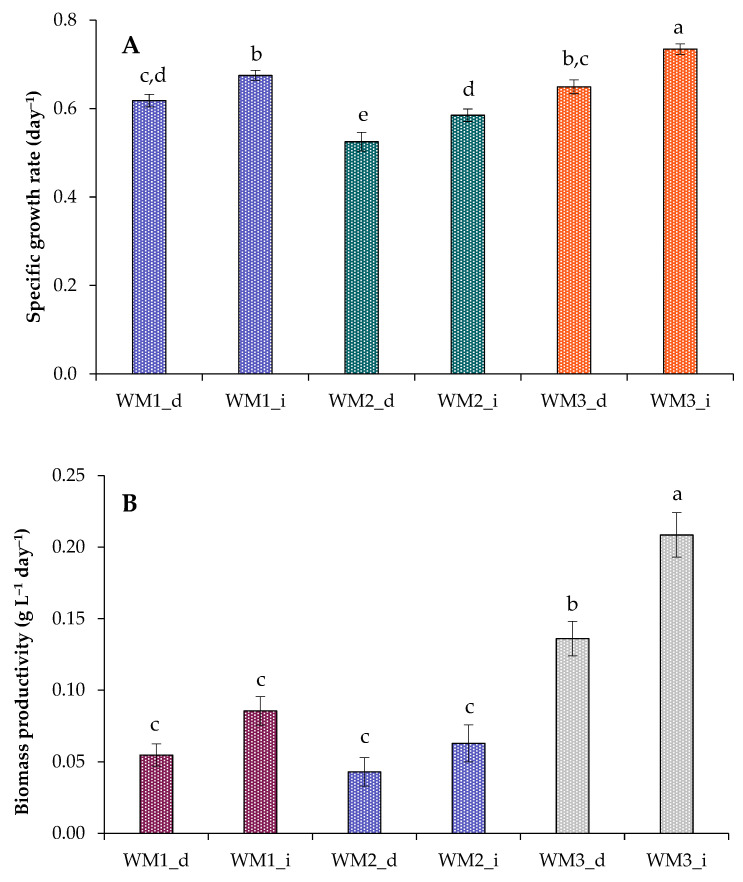
Specific growth rate (**A**) and biomass productivity (**B**) of *C. sorokiniana* grown in media based on wastewater (WM_d—diluted wastewater; WM_i—initial wastewater). Means that do not share a letter are significantly different.

Specific growth rate and biomass productivity were further calculated to describe the culture’s growth characteristics in wastewaters (Figure 5A,B). Thus, the highest specific growth rate of the culture was found in the WM3_i treatments (0.73 ± 0.01 day^−1^). The highest productivity of biomass was also noted during growth in undiluted wastewater samples from the third septic tank (0.21 ± 0.02 g L^−1^ day^−1^; Figure 5).

Fallahi et al. [40] characterized the growth characteristics of three microalgae species (*Chlorella vulgaris*, *Tetradesmus obliquus*, and *Nannochloropsis* sp.) in municipal wastewater and evaluated the growth parameters of a mixed culture. The maximum values of biomass concentration and biomass productivity were obtained for a mixed culture and amounted to 1.42 ± 0.15 g L^−1^ and 0.19 ± 0.01 g L^−1^ day^−1^, respectively. Separately, these values for *C. vulgaris* were 1.05 ± 0.01 g L^−1^ and 0.13 ± 0.01 g L^−1^ day^−1^, respectively [40]. These data are consistent with the results received in our work for WM3 treatments but for another algal species. In another work, mixotrophic cultivation of *C. sorokiniana* in primary treated domestic wastewater led to a biomass productivity of 0.08 g L^−1^ day^−1^ [41]. These results correspond to the values obtained in our study for WM1_i and WM2_i treatments, for which the biomass productivity was in the range of 0.06–0.09 g L^−1^ day^−1^. In a study performed by Gupta et al. [42], the values of the specific growth rate and biomass productivity of *C. vulgaris* when cultivated in municipal wastewater were 0.07 day^−1^ and 0.03 g L^−1^ day^−1^, respectively, which are lower than the values obtained in our study. To optimize cultivation, the authors proposed the addition of organic carbon sources, which made it possible to increase the efficiency of the process. This points to the importance of a sufficient amount of nutrients in wastewater to maintain physiological processes in microalgal cells. However, when increasing the productivity of microalgae by adding additional nutrients, special attention should be paid to the economic side of the strategy.

**Figure 6:**
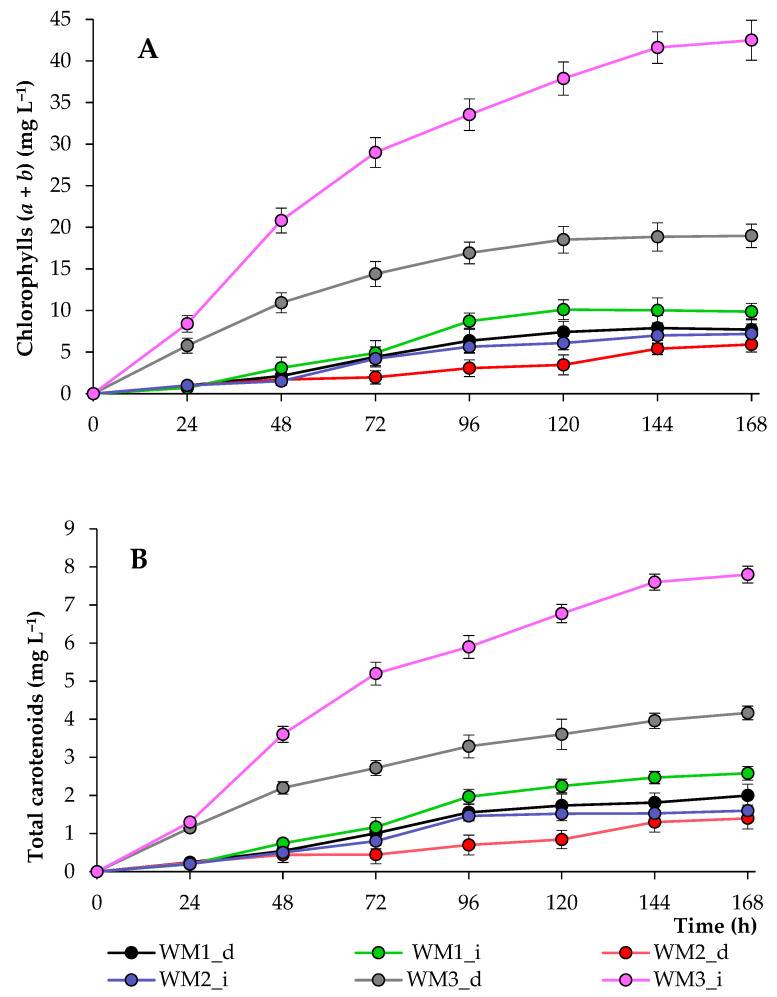
Chlorophyll (**A**) and carotenoid (**B**) concentrations of *C. sorokiniana* grown in media based on wastewater (WM_d—diluted wastewater; WM_i—initial wastewater).

The production of value-added products, including pigments, proteins, lipids, and carbohydrates, increases the economic efficiency of biomass production when wastewater is used as a growth medium [43]. The content of chlorophylls and carotenoids also confirmed that the wastewater from the third source had the most optimal parameters for algal growth. The maximum concentrations of chlorophylls in WM3_i and WM3_d samples were 42.5 ± 2.4 and 19.0 ± 1.4 mg L^−1^, respectively. In other treatments, chlorophyll concentrations did not exceed 10 mg L^−1^ (Figure 6).

The values obtained in this research were higher compared with the concentrations of pigments obtained when *C. vulgaris* was cultured in municipal wastewater (3.92 ± 0.8 mg L^−1^ of total chlorophylls) [40]. In another study, during the cultivation of *Chlamydomonas debaryana* in different wastewaters, the mean total pigment content was in the range of 10.5–12.4 mg L^−1^ [44], while the consortium of microalgae consisting of *Chlorella* sp. and *Scenedesmus* sp., when grown in 75% wastewater, produced about 27 mg L^−1^ of chlorophylls [45]. In a study performed by Sharma et al. [46], other consortia of microalgae were cultured in diluted municipal wastewater, and the highest mean chlorophyll concentration (25.2 mg L^−1^) was observed. *Scenedesmus acuminatus* culture accumulated the highest concentration of chlorophyll *a* and chlorophyll *b* (59.9 ± 3.5, 18.4 ± 3.6 mg L^−1^, respectively) when cultivated in 50% synthetic municipal wastewater, while the maximum concentration of carotenoids was obtained in 75% and 100% synthetic wastewater (20.1 ± 0.4 mg L^−1^ and 19.8 ± 0.1 mg L^−1^, respectively) [47]. The authors noted that an increase in the concentration of wastewater increases the stress index, which may explain the intense accumulation of carotenoids in highly concentrated nutrient media. In our previous work [4], it was also noted that *C. sorokiniana and C. vulgaris* accumulate carotenoids more intensively with an increase in the concentration of liquid waste in the growth medium.

**Figure 7:**
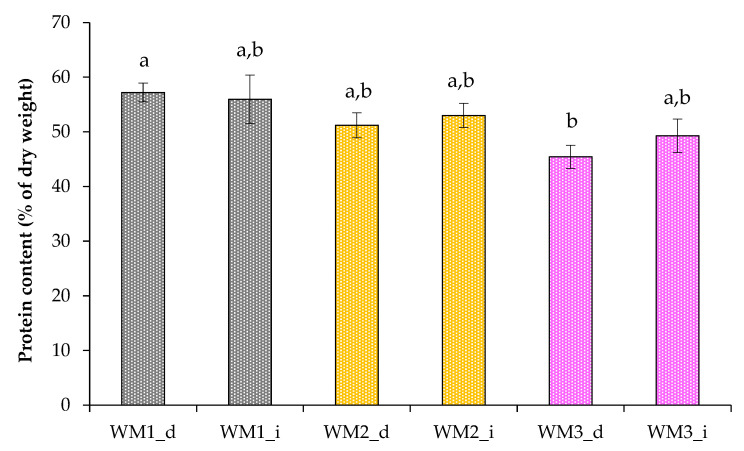
Protein content in *C. sorokiniana* cells grown in wastewater (WM_d—diluted wastewater; WM_i—initial wastewater). Means that do not share a letter are significantly different.

The biomass of *C. sorokiniana* grown in wastewater was rich in protein, the average content of which varied from 45.4% to 57.2% of dry weight (Figure 7). These values exceeded those obtained when grown in a modified BBM (Figure 3 and Figure 7). However, microbial communities that grew in unsterilized domestic wastewater with alga could make an additional contribution to the obtained biomass and protein data.

A study performed by Lu et al. [48] also demonstrated the high protein content in *Chlorella* sp. cells cultivated in mixed wastewater, which was 60.9–68.7%. In another work [49], *A. pyrenoidosa* that was cultivated mixotrophically in wastewater with a high content of NH_4_^+^ also had a high protein level (56.7% of dry weight). The results of recent work performed by Lavrinovičs et al. [50] showed that phosphorus deficiency in the nutrient medium increased protein accumulation and the rate of phosphorus uptake by the *C. vulgaris* culture, which can be considered a strategy for advanced wastewater treatment. Arrojo et al. [51] observed the maximum protein content in the biomass of *Chlorella fusca* (50.5%) when grown in 75% wastewater. As a result of continuous cultivation of *C. sorokiniana* in mixed industrial and municipal wastewater in lab-scale flat-panel photobioreactors, the average yield of protein was 0.39 g per gram of dry biomass [43]. When using poultry slaughterhouse wastewater as a culture medium for *Neochloris* sp., the maximum protein content was 41.7% [52]. In another study, Michelon and colleagues [53] tested *Chlorella* spp. under conditions simulating pig wastewater and noted the possibility of obtaining biomass with a high protein content (~50%). In our previous work [4], it was shown that microalgal cells of the families *Chlorellaceae* and *Scenedesmaceae* increased their protein content with an increase in the concentration of anaerobic digester effluent in the growth medium.

**Figure 8:**
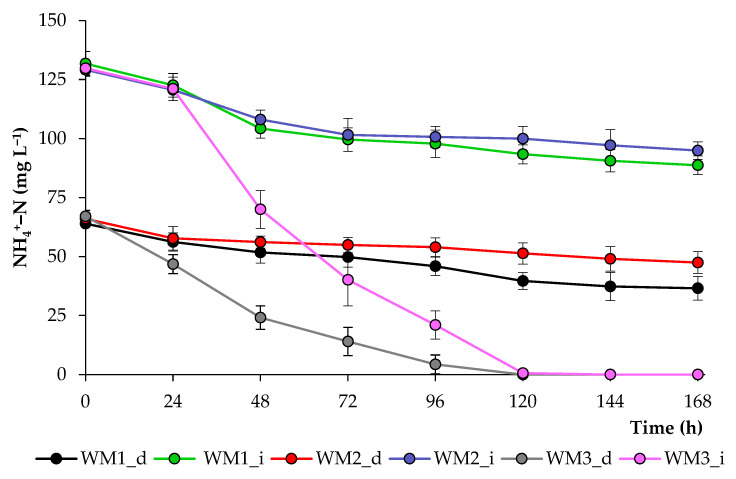
Ammonium nitrogen concentrations in media based on wastewater during the growth of *C. sorokiniana*.

To introduce microalgae into biological water treatment systems, it is important to evaluate the efficiency and rate of contaminant removal. Domestic wastewater may contain various sources of nitrogen, including ammonia, ammonium, nitrite, nitrate, and N-containing organic compounds [54]. Nitrogen removal trends are presented based on ammonium concentrations in the growth media (Figure 8). In all initial wastewaters, the mean NH_4_^+^–N concentration was adjusted to 130 mg L^−1^, and in diluted wastewaters, it was two times less. In treatments WM1 and WM2, the concentration of NH_4_^+^–N changed insignificantly. In the WM3_d treatment, NH_4_^+^–N was almost completely removed after 96 h, while in the WM3_i treatment, 100% of the NH_4_^+^–N was removed at 120 h. Since the mechanisms of nutrient removal are interrelated [54,55], the limited intake of one or another macro- or microelement may be associated with a deficiency of other components of the nutrient medium. Also, wastewater may contain too high concentrations of certain contaminants that inhibit the growth of the algal culture. Gao et al. [56] found that the proportion of NH_4_^+^–N in filtered domestic wastewater is more than half of the total nitrogen load, and nitrites and nitrates make up an insignificant proportion of nitrogen compounds. This indicates that ammonium removal rate is an important indicator of the optimal growth of algal cultures in domestic wastewater.

The cultivation of microalgae in wastewater is a promising way to reduce the cost of biomass production as well as an additional way to treat contaminated waters [16]. The optimal conditions for the growth of algae in wastewaters depend on the type of wastewater, pretreatment, collection time, and a few other factors [9,57]. While the composition of standard synthetic media is mostly balanced and constant, wastewater is characterized by a variable composition and bacterial contamination, which affects the parameters of the medium [9]. Domestic wastewater has an advantage over other types of wastewaters as it has lower concentrations of pollutants, including nitrogen, phosphorus, and various organic substances [58]. However, the presence of a large number of pathogenic bacteria in this type of waste requires some pretreatment to reduce the bacterial load [55]. Ramsundar et al. [41] assessed the impact of domestic wastewater pretreatment methods on the productivity of *C. sorokiniana* and the efficiency of pollutant removal. The authors reported that wastewater from primary treatment and anaerobic treatment has the most favorable nutritional composition but contains a significant number of microorganisms. When comparing methods for reducing the microbial load, it was found that filtration treatment is the most accessible and effective method compared to autoclaving, which changes the physical and chemical properties of wastewater [41]. Since autoclaving on an industrial scale is not a cost-effective treatment method, domestic wastewater was treated by filtration in the present study.

Overall, the results obtained show that the combination of continuous lighting and balanced nutrition can lead to the highest final biomass yield, highlighting the possibility of obtaining more biomass without replacing large volumes of nutrient medium. The dilution of wastewater allowed for a decrease in the bacterial load in cultivation systems but decreased the amount of biomass and value-added products, probably due to a lack of micronutrients for efficient algal cell growth. The dilution of wastewater will ultimately increase the cost of the bioremediation process.

### 2.3. Bacterial Community Associated with Wastewaters

In the wastewater treatment industry, much attention is paid to the analysis and monitoring of microbial communities, especially pathogenic microorganisms. Separately, the microbial communities of wastewater treatment systems that use microalgae are studied. Bacterial partners can have different types of relationships with algae in the culture medium. The study of beneficial interactions in bacterial–algal consortia is important for developing a strategy for more complete wastewater treatment from pollutants [27]. However, if the goal of cultivation is not only wastewater treatment but also the maximum accumulation of algal biomass, it is necessary to control the bacterial load.

Depending on the location and economic activity of the population, urban wastewater has a different microbial load and may also contain pathogenic microorganisms. It is reported that conditions for the cultivation of microalgae have a significant impact on the development of bacteria in wastewater. Several factors affecting the removal of individual bacteria from algal-bacterial systems are aeration, the addition of carbon dioxide, and the photoperiod. For example, these factors affected the elimination of sanitary-indicative microorganisms from domestic wastewater (such as *Pseudomonas aeruginosa*, *Enterococcus faecalis*, and *Escherichia coli*) [59].

In this study, traditional culture methods of microbiology were used to identify bacteria in the original wastewaters. Bacterial isolates from the three household wastewaters used in the work were obtained on a nutrient agar medium. The bacterial isolates were then grouped using restriction fragment length polymorphism analysis of the 16S rRNA gene, and the taxonomic identity of representative isolates was confirmed by sequencing their 16S rRNA gene fragments. About 200 bacterial isolates were obtained, and the 16S rRNA gene sequences of 20 isolates were determined (Table 3).

All bacterial 16S rRNA gene sequences were assigned to the phylum *Proteobacteria* (*Pseudomonadota*). It should be noted that representatives of the class *Gammaproteobacteria* accounted for the majority of cultivated bacterial communities. The 16S rRNA gene sequence of bacterial isolate WW117 had low matches in the nucleotide database, and this isolate may represent a new bacterial species (Table 3). An analysis of the 16S rRNA gene made it possible to identify the main representatives of the bacterial community, namely members of the genera *Acinetobacter*, *Pseudomonas*, *Aeromonas*, *Phyllobacterium*, *Stenotrophomonas*, and *Escherichia*. Despite the collection of water from different septic tanks, the wastewater was characterized by similar bacteria but in different proportions.

Urban infrastructure creates conditions for the development of certain microbial communities in wastewater, which is rich in human waste. Representatives of the genera *Pseudomonas*, *Acinetobacter*, and *Aeromonas* are found in urban wastewaters and are capable of metabolizing various carbon and nitrogen compounds [60,61]. For example, *Acinetobacter* sp., isolated from municipal activated sludge, was able to actively remove inorganic nitrogen compounds by simultaneously performing nitrification and denitrification [62], similar to *Pseudomonas* spp., which is also actively involved in the nitrogen cycle [63].

Bacteria of the genus *Stenotrophomonas* are involved in bioremediation processes [64,65] and are also important partners for plants [66]. The *Stenotrophomonas maltophilia* identified in this work is the most typical representative of this genus. This species is characterized by phenotypic diversity and, therefore, is adaptive to various ecological niches. *S. maltophilia* produces a wide range of biologically active compounds, including exoenzymes that inhibit the growth of pathogenic bacteria and fungi. The genome of *S. maltophilia* ZBG7B contains genes responsible for the synthesis of enzymes useful in the process of biodegradation, namely xylosidases, xylanases, laccases, and chitinases [67]. Thus, bacteria of the genus *Stenotrophomonas* have great potential for application in biotechnology and can act as useful partners for microalgae in wastewater treatment. However, some bacteria of this genus pose a threat to human health [68].

In a recent study [69], it was shown that wastewater treatment by microalgae of the genus *Coelastrella* resulted in a decrease in the number of pathogenic bacteria. A validation test performed by co-culturing microalgae and pathogenic bacteria of the genus *Oligella* (*Betaproteobacteria*) and a further network analysis showed that the decrease in the number of pathogens in wastewater was not a direct effect of *Coelastrella* sp. but was caused by bacteria associated with microalgae, namely *Brevundimonas*, *Sphingopyxis*, and *Stenotrophomonas*. In addition, microalgal metabolic products, which have a bactericidal effect, have a great impact on the bacterial communities in wastewater. For example, a number of studies have shown that microalgae are capable of producing a mixture of polyunsaturated fatty acids with antibiotic activity against various bacteria [70,71]. However, bacteria, in turn, can also affect the growth of microalgae through competition for nutrients [72,73]. Thus, further studies are needed, including under conditions of continuous cultivation of microalgae, which will reveal the possibility of the participation of different microalgal species in the treatment of other wastewaters.

## 3. Materials and Methods

### 3.1. Inoculum Preparation

The green alga *Chlorella sorokiniana* AM-02 was isolated from a water reservoir in Kazan (Republic of Tatarstan, Russia) and identified as described earlier by Ziganshina et al. [17]. A culture was maintained on the standard BBM (with sodium nitrate as a N source) solidified with agar [74]. Ampicillin and kanamycin were added to standard BBM to reduce the risk of bacterial contamination (10 µg and 50 µg per 1 mL of medium, respectively). Five days before the start of the experiments, individual colonies of *C. sorokiniana* were added under aseptic conditions to sterile 250 mL Erlenmeyer flasks containing 30 mL of sterile standard BBM and cultivated in a shaker at 120 rpm at 30 °C. One LED full spectrum lamp (ULI-P10-18W/SPFR, Uniel, China) was used as a light source. The accumulated biomass was centrifuged at 4000× *g* for 5 min, washed with sterile phosphate buffer (Na_2_HPO_4_ + KH_2_PO_4_, pH 7.0), and the inoculum was sterilely transferred into a photobioreactor to reach a final OD_750nm_ of 0.05 (optical density measured at 750 nm).

### 3.2. Experimental Conditions in the Reactor

Several parameters for the photoautotrophic growth of the tested microalga were optimized earlier [17,27,75]. The cultivation of microalga in various modes was carried out in a 6.8 L autoclavable borosilicate glass culture cylindric vessel with a working volume of 5.0 L (BIOSTAT A-plus, Sartorius, Germany). LED lamps (ULI-P10-18W/SPFR, China) were used as a light source and evenly distributed vertically around the vessel. The light intensity (800 µmol photons m^−2^ s^−1^ (measured on the surface of the vessel)) was measured using a photosynthetically active radiation (PAR) meter (Apogee Instruments, Logan, UT, USA). The algal strain was cultivated at a temperature of 30 °C and at a stirring speed of 100 rpm. The temperature inside the vessel was controlled with an automatic cooling water control valve (during the light period) and with a heating blanket (during the dark period). Aeration (1.3 L min^−1^) was provided using a compressor through a 0.20 µm Midisart 2000 filter with a PTFE (autoclavable) membrane (Sartorius Stedim Biotech, Göttingen, Germany). A digital mass flow controller/mass flow meter SmartTrak 50 (Sierra Instruments, Monterey, CA, USA) was used to add CO_2_ into the vessel (air and CO_2_ were mixed before being added to the vessel; the final supply of 2.0% CO_2_ was reached). The flow of CO_2_ was measured using a gas analyzer (Infors HT, Bottmingen, Switzerland). Sterile 2% foam suppressant (Antifoam B, Sigma-Aldrich, St. Louis, MO, USA) was added to prevent foaming. The pH was measured with an EasyFerm Plus PHI K8 425 electrode (Hamilton, OH, USA) and maintained at 7.0 ± 0.05 with the automatic addition of a sterile 2% HCl or 8% NaOH solution.

### 3.3. Cultivation Modes

The experiments were divided into two main blocks: (1) cultivation of *C. sorokiniana* in modified synthetic media and (2) cultivation of *C. sorokiniana* in domestic wastewater. 

Photoautotrophic cultivation of the microalgal strain was carried out in a sterile modified nutrient medium (BBM) with different contents of NH_4_^+^–N (180–360 mg L^−1^), PO_4_^3−^–P (53–80 mg L^−1^), and SO_4_^2−^–S (12–36 mg L^−1^). NH_4_Cl, H_3_PO_4_, and H_2_SO_4_ were used to increase the N, P, and S levels in the media. It should be mentioned that at pH 7.0, phosphate ions were found in the forms of H_2_PO_4_^−^/HPO_4_^2−^. The study used both standard modes of complete (SM1–SM5) and fractional nitrogen nutrition (SM6). Thus, in addition to the increased nitrogen content in the growth medium, the strategy of fractional addition of ammonium ions into the growth medium was evaluated (to decrease the possibility of ammonium toxicity to algal cells) (Table 1). In SM6 treatments, after the complete depletion of NH_4_^+^–N, the second portion of NH_4_^+^–N (180 mg L^−1^) was added to the medium (at 78 h of the experimental period). Culture growth was assessed both under a 16:8-h light/dark cycle and under continuous lighting (24 h).

As a source of nutrients in mixotrophic cultivation, domestic wastewater collected from three different septic tanks was used. Prior to cultivation of *C. sorokiniana*, wastewater samples were filtered with Whatman filter papers, and one part was then diluted with sterile distilled water to reach 50% (% *v/v*). Wastewater pre-treatment included the removal of undissolved solids and most microorganisms. Microalgal growth characteristics were evaluated at 50% and 100% wastewater concentrations. Since domestic wastewaters contained low concentrations of P and S, H_3_PO_4_ and H_2_SO_4_ were additionally added to the original wastewaters to achieve standard P and S concentrations, as in standard Bold’s Basal Medium [74]. NH_4_^+^–N levels were also adjusted to achieve the same concentrations (up to 130 mg L^−1^). The ammonium concentration was leveled with NH_4_Cl relative to the maximum detected in all wastewaters, namely up to 130 mg L^−1^. All these experiments were carried out under continuous lighting.

In all tested modes, microalga was cultivated until reaching the stationary growth phase, after which the final parameters were evaluated.

### 3.4. Algal Culture Growth, Productivity and Nutrient Removal Analyses

To assess the growth parameters, the optical density of the algal suspension was measured daily at 750 nm using a Lambda 35 spectrophotometer (Perkin Elmer, Singapore), and the number of cells was counted with a hemocytometer [4,75]. 

Chlorophylls and carotenoids (mg L^−1^) were determined in accordance with the methods described earlier [27,76]. 

At the end of each experiment, the biomass was concentrated using centrifugation at 5000× *g* for 3 min, washed twice with distilled water, and dried at 105 °C for 16 h. The final dry weight of the biomass (dry matter), as well as volatile solids, was estimated using an analytical balance [75,77]. 

The content of total ammonia nitrogen in the media was measured using Nessler’s reagent (Sigma-Aldrich, St. Louis, MO, USA) [75,78].

The concentrations of phosphate, sulfate, and nitrate in the growth media were evaluated using ion chromatography (Dionex ICS-900 Ion Chromatography System, Thermo Fisher Scientific, Wilmington, DE, USA) [17,79]. 

The content of proteins in dry algal biomass was estimated with the Bradford method [80]. About 20–30 mg of dried biomass at 60 °C was taken for sample analysis. Colorimetric measurement was performed using a Bio-Rad protein assay kit, as described previously [27].

The specific growth rate and biomass productivity were estimated as described by Nayak et al. [81].

Each experiment was conducted in duplicate, all analyses were measured in triplicate, and the mean values are presented together with standard deviations.

The Tukey method and 95% confidence were used to compare differences (Minitab software version 20.2.0, State College, PA, USA).

### 3.5. Bacterial Community Structure Analysis

Bacterial isolates were obtained from original wastewaters on nutrient agar plates and then cultured at 30 °C for 24–48 h. Bacterial 16S rRNA gene fragments were amplified using PCR with the primers UniBac27f (5′-AGA GTT TGA TCM TGG CTC AG-3′) and Univ1492r (5′-TAC GGY TAC CTT GTT ACG ACT T-3′). About 200 bacterial colonies were collected and screened in PCR reactions. The library was then analyzed for restriction fragment length polymorphisms using the restriction endonucleases *Hae*III and *Rsa*I (Thermo Fisher Scientific, Vilnius, Lithuania). Restriction patterns were clustered with the Phoretix 1D software v. 16.2 (Nonlinear Dynamics, Newcastle upon Tyne, UK). The 16S rRNA genes of representative bacterial isolates from each main cluster were partially sequenced. The sequences were compared to the NCBI database using the BLAST program and classified according to the EzBioCloud Database [82].

## 4. Conclusions

This study employed strategies to improve the growth and productivity parameters of the indigenous freshwater microalga *Chlorella sorokiniana* in synthetic media and domestic wastewaters. The results showed that the growth of microalga in synthetic media with corrected levels of nitrogen, phosphorus, and sulfur led to an increase in the total biomass yield (up to 5.9 g L^−1^), content of chlorophylls and carotenoids (up to 180 and 26 mg L^−1^, respectively), and protein (up to 36% of the dry weight). Moreover, the correction of macronutrient levels in the growth media based on domestic wastewater allowed to produce biomass with a high protein content (45–57% of the dry weight). The results, based on a bacterial 16S rRNA gene sequencing approach, showed that most of the bacteria in the original wastewaters belonged to the class *Gammaproteobacteria*. Overall, the results obtained show a promising application of the tested microalga in the production of biomass and value-added products, including pigments and proteins.

## Figures and Tables

**Table 1:** Experimental design and biomass values obtained during cultivation of *C. sorokiniana* in synthetic media (SM) under different regimens (1–6).

Treatment	Light/Dark Period (h)	NH_4_^+^–N (mg L^−1^)	PO_4_^3−^–P (mg L^−1^)	SO_4_^2−^–S (mg L^−1^)	Dry Weight (g L^−1^)	Volatile Solids (g L^−1^)
SM1	16/8	180	53	12	3.17 ± 0.13 ^c^	2.83 ± 0.12 ^c^
SM2	24/0	180	53	12	4.17 ± 0.14 ^b^	3.85 ± 0.15 ^b^
SM3	24/0	180	80	36	4.40 ± 0.17 ^b^	4.10 ± 0.08 ^b^
SM4	24/0	360	53	12	4.61 ± 0.16 ^b^	4.13 ± 0.09 ^b^
SM5	24/0	360	80	36	5.93 ± 0.11 ^a^	5.33 ± 0.08 ^a^
SM6	24/0	180 + 180	80	36	5.68 ± 0.18 ^a^	5.13 ± 0.12 ^a^

Different superscripts indicate differences between the treatments (ANOVA, Tukey method, α = 0.05). Means that do not share a letter are significantly different.

**Table 2:** Biomass values obtained during cultivation of *C. sorokiniana* culture in three types of wastewaters.

Treatment	Dry Weight (g L^−1^)	Volatile Solids (g L^−1^)
WM1_d	0.28 ± 0.04 ^c^	0.23 ± 0.02 ^d,e^
WM1_i	0.43 ± 0.05 ^c^	0.35 ± 0.01 ^c^
WM2_d	0.22 ± 0.05 ^c^	0.18 ± 0.02 ^e^
WM2_i	0.32 ± 0.04 ^c^	0.26 ± 0.01 ^d^
WM3_d	0.82 ± 0.06 ^b^	0.69 ± 0.02 ^b^
WM3_i	1.26 ± 0.07 ^a^	1.06 ± 0.02 ^a^

Different superscripts indicate differences between the treatments (ANOVA, Tukey method, α = 0.05). Means that do not share a letter are significantly different. WM_d—diluted wastewater; WM_i—initial wastewater.

**Table 3:** Sequencing results of 16S rRNA genes of representative bacterial isolates.

Isolate (bp)	Highest BLAST Hit (Acc. No.)/ Percent Identity	Taxonomic Affiliation
WW1 (922)	*Acinetobacter lwoffii* ex19 (KF317889)/99.9%	*Acinetobacter* sp.
WW2 (932)	*Pseudomonas putida* B33 (KT767698)/99.9%	*Pseudomonas* sp.
WW55 (823)	*Pseudomonas* sp. H8 (MH885482)/100%	*Pseudomonas* sp.
WW94 (824)	*Pseudomonas* sp. JCM 5482 (AB685687)/99.9%	*Pseudomonas* sp.
WW8 (822)	*Pseudomonas* sp. CmLB7 (HM352331)/99.9%	*Pseudomonas* sp.
WW39 (857)	*Pseudomonas mendocina* SM5 (JX102498)/99.8%	*Pseudomonas* sp.
WW19 (876)	*Pseudomonas oleovorans* JCM 13980 (LC508006)/99.9%	*Pseudomonas* sp.
WW117 (802)	*Pseudomonas paralactis* strain DSM 29164 (NR_156987)/97.1%	*Pseudomonas* sp.
WW3 (799)	*Aeromonas salmonicida* HA-1 (OQ983639)/100%	*Aeromonas* sp.
WW14 (612)	*Phyllobacterium myrsinacearum* 608 (MT199181)/99.8%	*Phyllobacterium* sp.
WW23 (612)	*Phyllobacterium myrsinacearum* NBRC 100019 (NR113874)/100%	*Phyllobacterium* sp.
WW64 (684)	*Stenotrophomonas maltophilia* cqsm_h3 (MN826555)/99.7%	*Stenotrophomonas* sp.
WW103 (869)	*Stenotrophomonas maltophilia* cqsG6 (MN826545)/99.7%	*Stenotrophomonas* sp.
WW12 (838)	*Stenotrophomonas maltophilia* cqsG6 (MN826545)/100%	*Stenotrophomonas* sp.
WW74 (897)	*Stenotrophomonas maltophilia* S7-1 (MN732994)/99.8%	*Stenotrophomonas* sp.
WW11(839)	*Stenotrophomonas tumulicola* cqsG1 (MN826536)/99.6%	*Stenotrophomonas* sp.
WW13 (787)	*Stenotrophomonas chelatiphaga* P4M85 (MN421410)/99.6%	*Stenotrophomonas* sp.
WW105 (851)	*Stenotrophomonas* sp. V10R15 (MT165571)/100%	*Stenotrophomonas* sp.
WW18 (769)	*Escherichia coli* MAK15 (OP060224)/99.8%	*Escherichia coli*
WW44 (802)	*Escherichia coli* LCU-ID-EC1 (OL677623)/99.9%	*Escherichia coli*

## Data Availability

The data presented in this study are available on request from the corresponding author.
